# Laparoscopic Resection of an Unruptured Aneurysm of the Right Gastric Artery

**DOI:** 10.3400/avd.cr.24-00091

**Published:** 2025-02-12

**Authors:** Keiichiro Kawamura, Munetaka Hashimoto, Hiroko Sato, Shinichiro Horii, Atsumi Kosaka, Yoshihisa Tamate, Yuji Goukon

**Affiliations:** 1 Department of Vascular Surgery, Iwate Prefectural Isawa Hospital, Oshu, Iwate, Japan; 2 Division of Vascular Surgery, Department of Surgery, Soma General Hospital, Soma, Fukushima, Japan; 3 Department of Cardiovascular Surgery, Sendai City Hospital, Sendai, Miyagi, Japan

**Keywords:** gastric artery, aneurysm, laparoscopic surgery

## Abstract

We report a case of a 68-year-old female patient with an 8-mm right gastric artery aneurysm. The attempt at endovascular treatment was unsuccessful and therefore the patient underwent laparoscopic surgery for the resection of the unruptured right gastric artery aneurysm. The postoperative course was uneventful, and the patient is currently under observation as an outpatient. Although endovascular treatment is the first choice for treatment of unruptured right gastric aneurysms, laparoscopic surgery offers advantages such as less invasiveness, anatomical accessibility, and the ability to perform histopathological examination, making it an effective treatment option when endovascular treatment is difficult.

## Introduction

Visceral artery aneurysms (VAAs) are rare, with an estimated prevalence of approximately 1%.^1)^ Right gastric aneurysms are even rarer, accounting for less than 4% of all VAAs.^2)^ Although the number of reported cases of VAAs has been increasing in recent years with advances in diagnostic imaging technology, VAAs are often detected upon rupture due to a lack of subjective symptoms, with rupture being associated with a high mortality rate.^2)^ Treatment options for VAAs include open, laparoscopic, endovascular, or hybrid treatment,^3)^ depending on hospital facilities, aneurysm condition, and patient background. Herein, we report a case of an unruptured right gastric aneurysm that was discovered incidentally and treated laparoscopically. Endovascular surgery was attempted without success; therefore, the patient was shifted to laparoscopic surgery. Laparoscopic surgery for the right gastric aneurysm was considered an appropriate and safe procedure. The patient provided written informed consent for the publication of her case details and imaging studies.

## Case Report

**Figure 1:**
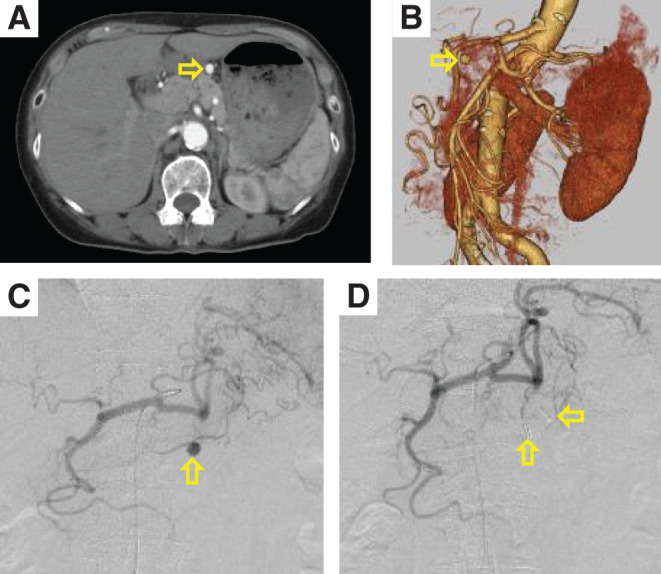
Fig. 1 Preoperative CT and intraoperative angiography. (**A**) Sagittal view of the right gastric artery measuring 8 mm (arrow). (**B**) Three-dimensional reconstructed CT image of the right gastric artery (arrow). (**C**) Angiography of the celiac artery showing the right gastric artery (arrow). (**D**) The aneurysm in the right gastric artery was completely resected. Arrows indicate the clips on the inflow and outflow arteries. CT: computed tomography

A 68-year-old female patient was referred to our hospital after an abdominal tumor was detected on ultrasonography. The patient had hypertension, for which she was receiving medical treatment, and a history of thalamic hemorrhage. There was no family history of aneurysm, evidence of smoking, trauma, pancreatitis, and concomitant risk factors such as dyslipidemia, diabetes mellitus, ischemic heart disease, or chronic kidney disease. Laboratory test results showed no elevated levels of inflammatory markers, with a leukocyte count and C-reactive protein level of 2480/µL and 0.22 mg/dL, respectively. In addition, there was no evidence of autoimmune disease, vasculitis, or fungal infection. A contrast-enhanced computed tomography (CT) revealed an 8-mm right gastric artery (**Figs. 1A** and **1B**). In this case, we decided to perform a laparoscopic aneurysmectomy if endovascular treatment was unsuccessful and to conduct the procedure under general anesthesia.

**Figure 2:**
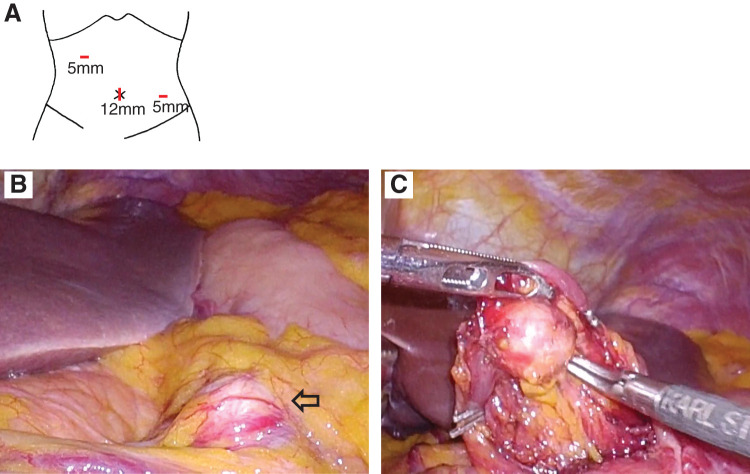
Fig. 2 Intraoperative findings during laparoscopic surgery. (**A**) Schema of port location during laparoscopic surgery. (**B**) The right gastric artery aneurysm (arrow) was easily identified. (**C**) Inflow and outflow arteries of the aneurysm were clipped using 5-mm clips.

**Figure 3:**
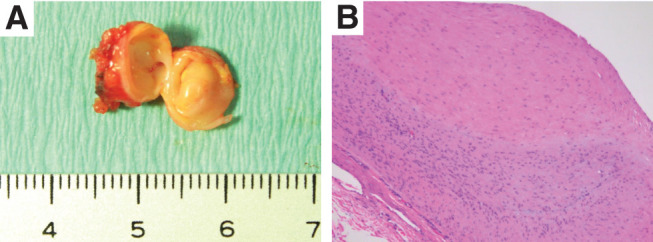
Fig. 3 Pathological insights. (**A**) Macroscopic examination of resected aneurysm. (**B**) Hematoxylin and eosin staining (100x) revealed only the formation of atheroma and thinning of the tunica media.

With the patient under general anesthesia, a 4Fr sheath was inserted into the right femoral artery, and angiography of the celiac artery was performed, showing a right gastric artery aneurysm (**Fig. 1C**). Cannulation from the proper hepatic artery to the right gastric artery was attempted using a 0.014-inch guidewire and a microcatheter. Although the guidewire and microcatheter could be inserted only into the gastroduodenal artery, the cannulation from the proper hepatic artery to the right gastric artery was difficult because of strong flexion. Therefore, endovascular treatment was deemed unfeasible, and the patient was converted to laparoscopic surgery. A 12-mm trocar was inserted through the umbilicus, and two additional 5-mm trocars were inserted into the right and left upper quadrants (**Fig. 2A**). The right gastric artery aneurysm was easily identified (**Fig. 2B**). The inflow and outflow arteries of the aneurysm were clipped with 5-mm clips (**Fig. 2C**), and the aneurysm was excised. Since angiography was possible with the sheath and catheter used for endovascular treatment, a final angiography was performed and complete resection of the right gastric artery aneurysm was confirmed (**Fig. 1D**). Surgical time was 37 min for endovascular treatment and 59 min for laparoscopic surgery, with minimal blood loss. The postoperative course was uneventful. The patient resumed eating and walking the day after surgery and was discharged on postoperative day 4. The histopathological diagnosis revealed only the formation of atheroma and thinning of the tunica media, while no fibromuscular dysplasia or segmental arterial mediolysis (SAM) was observed (**Fig. 3**).

## Discussion

VAAs are rare, often asymptomatic, and detected incidentally; however, they carry the risk of rupture.^1–3)^ Therefore, early diagnosis and appropriate treatment are essential. The number of reported cases of VAAs has increased in recent years because of advances in imaging technology, which has allowed clinicians to diagnose all types of VAAs, such as aneurysms of the celiac artery, superior mesenteric artery, inferior mesenteric artery, and their branches.^3)^ Stanley et al. reported the distribution of aneurysm sites as follows: 60% in the splenic artery, 20% in the hepatic artery, 5.5% in the superior mesenteric artery, 4% in the celiac artery, 4% in the gastric and gastroepiploic artery, 3% in the small intestinal and colonic artery, 2% in the pancreaticoduodenal artery, 1.5% in the gastroduodenal artery, and less than 1% in the inferior mesenteric artery.^2)^

Causes of VAAs include arteriosclerosis, congenital, hyperplasia, portal hypertension, inflammation, infection, trauma, arteritis, and pregnancy. In the present case, although tests for vasculitis and genetic conditions were not performed, these conditions were ruled out based on clinical findings. In addition, with recent advances in diagnostic imaging, SAM has been attracting attention. SAM, first described by Slavin and Gonzalez-Vitale in 1976, is a disease in which the arterial media undergo segmental fusion to form aneurysms, occurring not only in a single arterial system but also in multiple arteries.^4)^ In the present case, the aneurysm was single, and the histopathological diagnosis showed only atheroma formation along with thinning of the arterial media, with no evidence of SAM. However, since some reports have diagnosed SAM based on clinical findings,^5)^ the possibility of a right gastric aneurysm due to SAM cannot be completely ruled out. Therefore, careful follow-up, including postoperative imaging studies, is considered necessary.

According to the Society for Vascular Surgery guidelines for VAAs, unruptured gastric aneurysms are indicated for treatment upon detection regardless of size.^6)^ VAAs can be treated via open surgery, laparoscopic surgery, endovascular therapy, or a hybrid approach,^3)^ in which endovascular treatment is recommended as the first-line treatment for all gastric artery aneurysms.^6)^ Furthermore, Barrionuevo et al. conducted a systematic review and meta-analysis, suggesting that endovascular treatment was associated with a shorter hospital stay and lower rates of cardiovascular complications.^7)^ On the other hand, open surgery remains the procedure of choice for rupture because it allows intraoperative confirmation of organ ischemia and provides resected specimens for pathological diagnosis.^8)^ Laparoscopic surgery has the advantages of being less invasive than open surgery and allowing histopathological examination compared to endovascular therapy. These advantages have been presented in case reports of laparoscopic surgery for right gastric aneurysms in both ruptured and unruptured cases. Toyoda et al. reported a favorable outcome following less invasive laparoscopic surgery for a ruptured right gastric aneurysm in a patient with stable circulatory dynamics.^9)^ Kimura et al. reported a case involving the combined laparoscopic resection of an unruptured right gastric aneurysm and a gastric gastrointestinal stromal tumor, which facilitated histopathological examination.^10)^ We considered laparoscopic surgery appropriate for a right gastric aneurysm, as in this case, especially due to its anatomical accessibility and the lack of need for revascularization given the well-developed arcades. In addition, the general condition of the patient was good, with no complications or history of abdominal surgery, which reduced the concern for intraperitoneal adhesion. Therefore, we decided to perform laparoscopic aneurysm resection as the second-line treatment when endovascular treatment was unsuccessful, since surgical treatment was considered anatomically feasible. Angiography before and after aneurysm resection allowed confirmation of the anatomic location after resection, and the postoperative course was uneventful. Angiography may not be standard in this case, as laparoscopic surgery was performed after endovascular treatment failed. However, we believe that angiography using the sheath and catheter from the endovascular treatment was appropriate because it allowed us to confirm that the aneurysm was completely resected and there was no bleeding intraoperatively. Laparoscopic surgery following the failure of endovascular treatment is a suitable and safe procedure for VAAs, including right gastric artery aneurysms. However, the optimal treatment strategy for VAAs remains controversial, and laparoscopic surgery may be an option for the treatment of VAAs.

## Conclusion

We report a case of an unruptured right gastric artery aneurysm that was successfully treated with laparoscopic surgery. VAAs are rare but potentially life-threatening. Therefore, early diagnosis and appropriate treatment are essential. Although endovascular treatment is the first choice for unruptured right gastric aneurysms, laparoscopic surgery offers the advantages such as less invasiveness, anatomical accessibility, and the ability to perform histopathological examination, making it an effective treatment option when endovascular treatment is difficult.

## Declarations

### Acknowledgments

We would like to thank Editage (www.editage.jp) for the English language editing.

### Informed consent

Informed consent was obtained from the patient to publish her details.

### Disclosure statement

All authors have no conflict of interest.

### Author contributions

Study conception: KK

Data collection: KK

Writing: KK, MH

Critical review and revision: all authors

Final approval of the article: all authors

Accountability for all aspects of the work: all authors.
